# Association Between the EAT-Lancet Reference Diet and Gestational Diabetes Mellitus: A Mini-Review

**DOI:** 10.3390/nu16234073

**Published:** 2024-11-27

**Authors:** Niuniu Sun, Shubo Wen, Zhenyu Huo, Zitong He, Tongyao Sun, Jingxi Hu, Emily Sonestedt, Yan Borné, Shunming Zhang

**Affiliations:** 1 School of Nursing, Henan University of Science and Technology, Luoyang 471023, China; 9905579@haust.edu.cn (N.S.); wenshubo972@163.com (S.W.); 2 School of Public Health, North China University of Science and Technology, Tangshan 063000, China; huozhenyu0512@163.com; 3 School of Public Health, Xi’an Jiaotong University Health Science Center, Xi’an 710061, China; hezitong1020@163.com (Z.H.); suntongyao0409@163.com (T.S.); hujingxi2028@163.com (J.H.); 4 Nutritional Epidemiology, Department of Clinical Sciences Malmö, Lund University, 21428 Malmö, Sweden; emily.sonestedt@med.lu.se (E.S.); yan.borne@med.lu.se (Y.B.)

**Keywords:** EAT-Lancet, diet, gestational diabetes mellitus, pregnancy

## Abstract

Gestational diabetes mellitus (GDM) is a prevalent pregnancy complication that seriously endangers maternal and infant health, posing a medical and economic burden worldwide. Several dietary patterns have been recommended for women of childbearing age, demonstrating a positive role in preventing and managing GDM. However, these dietary patterns may not fully take environmental factors into account when addressing global food sustainability and planetary health. In this context, the EAT-Lancet Commission proposed a diet in 2019 aimed at both health improvement and environmental sustainability, which can potentially reduce the prevalence of diet-related diseases. Nevertheless, the role of the EAT-Lancet reference diet in preventing and managing GDM has not been fully evaluated. Therefore, we conducted a literature search to assess the existing evidence for the association between the EAT-Lancet reference diet components and GDM. Based on the current evidence available in the PubMed database from inception to 31 October 2024, women of childbearing age are recommended to consume whole grains, fish, soy products, olive oil, full-fat dairy products, nuts, and moderate amounts of fruits while reducing red meat and sugar-sweetened beverage intake to lower the risk of GDM. There remains inconsistency regarding the association between tubers or starchy vegetables, vegetables, eggs, and poultry and the risk of GDM. In conclusion, current research on the association between diet and GDM is limited and offers suggestions for methodologies to obtain robust evidence regarding the association between the EAT-Lancet reference diet and GDM.

## 1. Introduction

Gestational diabetes mellitus (GDM) occurs when women without previous diabetes develop high blood sugar levels from disrupted glucose metabolism during pregnancy [1]. About 14% of pregnant women globally suffer from GDM [2]. Given its association with higher risks of adverse pregnancy outcomes and long-term metabolic issues for both mothers and infants [3], GDM has been identified as one of the primary challenges in improving maternal and infant health. Research has indicated that GDM was positively associated with chronic low-grade inflammatory disease [4,5]. Women with GDM have increased risks of various post-partum complications such as vascular dysfunction [6] and dyslipidemia [7]. Post-partum complications are mainly associated with inflammatory-induced endothelial dysfunction and insulin resistance [8]. Additionally, among women with a history of GDM, the lifetime risk of type 2 diabetes is higher than that of women without this condition, and the incidence rates of hypertension and ischemic heart disease are significantly increased [9]. For offspring whose mothers have had GDM, there is a higher risk of metabolic and cardiovascular complications [10]. Furthermore, maternal vascular diseases caused by GDM can lead to fetal oxygen deprivation, suffocation, and even stillbirth or miscarriage [11]. At the same time, there is evidence suggesting that maternal hyperglycemia without adequate management can adversely impact offspring neurodevelopment [12]. Also, GDM has imposed a huge economic burden worldwide [13,14,15].

Preventing GDM is critical for reducing both maternal and infant complications, as well as lessening its overall burden. Diet represents a primary adjustable risk factor for GDM [16]. Improving the diet of pregnant women with GDM can assist in stabilizing blood glucose levels, minimizing complications, and supporting postpartum recovery [17]. A healthy diet can lower the incidence of macrosomia and improve the prognosis for newborns [18]. Various dietary approaches, including the Mediterranean Diet [19] and the DASH Diet (Dietary Approaches to Stop Hypertension) [20], have been inversely associated with GDM. In addition, food production represents a significant driving force behind global environmental change [21].

**Figure 1:**
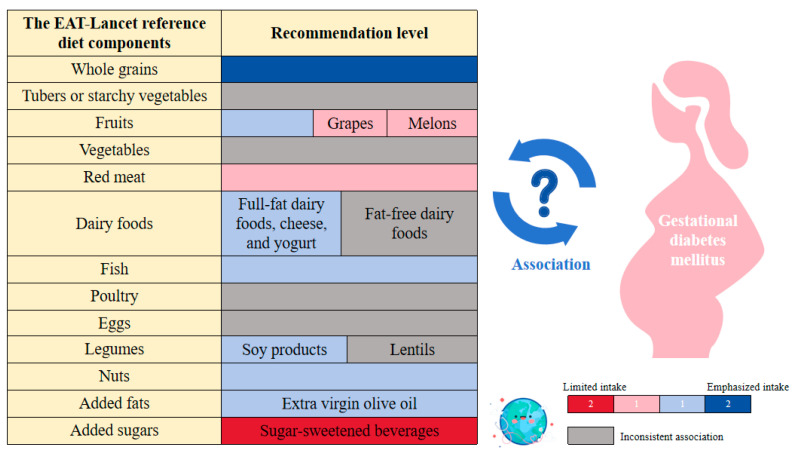
The relationship between the EAT-Lancet reference diet components and gestational diabetes mellitus in pregnant women (The icons are from https://www.freepik.com/).

Given the potential of the food system to enhance public health and foster environmental sustainability, a diet that benefits both planetary health and the prevention of GDM is extremely important. In this context, the reference diet, proposed by the EAT-Lancet committee, was launched in 2019 to provide a healthy diet for the increasing global population, simultaneously maintaining the potential of the food system to enhance public health and foster environmental sustainability [21]. This global reference diet advocates a plentiful consumption of plant-based foods, including whole grains, vegetables, fruits, legumes, nuts, and unsaturated oils. It supports a moderate intake of fish and poultry while advising a reduction in added sugar, dairy products, and red meat. The transition from a low-quality diet to the EAT-Lancet reference diet may reduce the global rates of diet-related diabetes [22,23]. This mini-review presents a narrative summary of the existing evidence, based on the literature in the PubMed database from inception to 31 October 2024, to assess the association of the EAT-Lancet reference diet components (including whole grains, tubers or starchy vegetables, fruits, vegetables, dairy foods, red meat, fish, poultry, eggs, legumes, nuts, added fats, and added sugars) with GDM (Figure 1).

## 2. The EAT-Lancet Reference Diet Components and GDM

### 2.1. Whole Grains

Several earlier investigations have indicated that maternal consumption of whole grains was associated with the incidence of GDM. For instance, a meta-analysis involving 13 cohort studies documented that whole grain intake assessed by the food frequency questionnaire and dietary recall methods showed an inverse association with GDM [24]. In another cohort study involving 15,632 women without a prior diagnosis of GDM as part of the Nurses’ Health Study II (1991–2001), it was indicated that replacing two servings of potatoes with whole grain foods per week was notably associated with a reduction in the incidence of GDM [25]. Furthermore, a cohort study performed in Iceland documented that increasing whole grain intake was associated with a reduced incidence of GDM [26]. Additionally, different grain processing methods significantly affected the absorption efficiency of their nutritional components and had varying impacts on GDM [26,27]. For example, a randomized controlled trial (RCT) indicated that among pregnant women, including those diagnosed with GDM as well as healthy individuals, the intake of sourdough whole grain wheat bread resulted in a 45.5% reduction in insulin secretion and a 9.6% decrease in blood glucose levels within the first hour after a meal, compared to the intake of white wheat bread [28].

Based on these cohort studies, it is evident that higher whole grain consumption is associated with a lower risk of GDM. Moreover, different processing methods may impact the relationship between whole grain intake and GDM.

### 2.2. Tubers or Starchy Vegetables

A few studies have been carried out to examine the association between tubers or starchy vegetables and GDM. For instance, a cohort study involving women with a singleton pregnancy of 8–14 weeks’ gestation found that a higher dietary glycemic load, particularly from starchy foods, showed an inverse association with GDM [29]. In contrast, another cohort study, which included women with both singleton and multiple pregnancies, assessed the total dietary intake of starchy vegetables and their subcategories, such as potatoes and other tuber vegetables (pumpkin, lotus root, yam, taro, water chestnut, pea, and cowpea). This study found that consuming more starchy vegetables and potatoes was associated with a higher risk of GDM, whereas the intake of other starchy vegetables showed no significant association with GDM [30]. Additionally, a study focused on women aged 18 to 45 years during pregnancy found no significant association between the intake of potatoes and other tuber vegetables and risk of GDM [31].

These inconsistencies among existing cohort studies hinder the establishment of a definitive association between the intake of tubers or starchy vegetables and GDM.

### 2.3. Fruits

Several previous studies have demonstrated that the maternal intake of fruits is associated with GDM. For example, a few RCTs have shown an inverse correlation between the consumption of fruit and the likelihood of developing GDM [32]. Consuming one cup of whole berries and one cup of leafy greens daily has been found to enhance the metabolic processes linked to the development and outcomes of GDM, which may contribute to the prevention of this condition [33]. Similarly, a case-control study indicated an association between following a fruit and vegetable diet during pregnancy and a lower occurrence of GDM, potentially associated with improvements in gut microbiota [34].

Additionally, because fruits contain natural sugars like fructose and sucrose, different levels of fruit consumption may have varying associations with GDM [35,36]. A cohort study showed that consuming a minimum of five servings of vegetables and fruits daily was associated with a lower risk of GDM [37]. Furthermore, a meta-analysis of 12 cohort studies, comprising a total of 32,794 participants, indicated that a daily increase of 100 g in fruit consumption was associated with a 3% decrease in the risk of GDM [38]. In contrast, the average intake of grapes and melons in the highest quartile, at 246 g and 297 g, respectively, was associated with elevated blood glucose levels and an increased risk of GDM, highlighting the need for moderate fruit consumption [39]. Moreover, adding more fruits and vegetables to the diet among pregnant women diagnosed with GDM has been associated with a reduced incidence of preterm delivery and infants with low birth weight [40].

Based on the existing evidence mentioned above, fruit intake is associated with GDM, and moderate fruit consumption not only reduces the incidence of GDM but is also associated with improved outcomes for pregnant women with this condition. However, the association between different types of fruits and GDM may vary, indicating that specific fruit consumption could have distinct impacts on GDM.

### 2.4. Vegetables

In the majority of existing studies, as described within the fruit food group, fruits and vegetables were often investigated together, and an inverse association with GDM was observed. Similarly, a case-control study indicated that a higher vegetable factor score was associated with a lower risk of GDM, regardless of whether the assessment was conducted in the year before conception or throughout the first and second trimesters of pregnancy [41]. However, a meta-analysis encompassing 12 cohort studies reported no association between increased vegetable intake and a reduced risk of GDM [38].

According to existing evidence, when fruits and vegetables are analyzed collectively, vegetable intake is inversely associated with the risk of GDM. However, few studies have investigated vegetable intake in isolation, and the existing evidence remains inconsistent, making it challenging to definitively ascertain the association between vegetable intake and GDM.

### 2.5. Dairy Foods

According to the 2020–2025 Dietary Guidelines for Americans, it was recommended that pregnant individuals consume three servings (one serving was defined as 240 milliliters of milk or yogurt, 42 g of hard cheese, or 28 g of soft cheese) of dairy products daily to help maintain a healthy weight and lower the risk of GDM [42]. In addition, the 2016 and 2022 editions of the Chinese Dietary Guidelines recommend that pregnant women consume 300–500 g of dairy products per day [43].

Several studies demonstrated an association between consuming dairy foods and GDM. In particular, consuming more dairy foods, especially full-fat options, was associated with a decreased risk of GDM [44,45]. Conversely, in a cohort study, individuals who consumed no fewer than three servings of dairy daily between the 24th and 28th weeks of gestation were categorized into three groups based on their intake of fat-free dairy foods (including skim milk, fat-free yogurt, and cheese). This study concluded that fat-free dairy consumption was not associated with the risk of GDM [46].

Additionally, both the consumption of cheese and probiotic yogurt have been inversely associated with the risk of GDM [47,48]. Notably, a RCT study indicated that the daily consumption of vitamin D3-enriched yogurt increased insulin sensitivity in pregnant women with GDM [49].

In summary, while there is evidence suggesting that full-fat dairy products may decrease the risk of GDM, the association between fat-free dairy products and GDM remains uncertain. Furthermore, cheese and yogurt are associated with a lower risk of GDM.

### 2.6. Red Meat

Several cohort studies have elucidated the association between red meat consumption and GDM. Research conducted in China and Spain demonstrated a significant association between elevated red meat intake and a higher risk of GDM [50,51]. Additionally, a cohort study involving 635 Iranian pregnant women found that the intake of red meat and processed meat was positively correlated with the risk of GDM [52]. Furthermore, a meta-analysis of 17 cohort studies revealed that each incremental increase of 100 g of red meat consumption daily was associated with a 1.94-fold elevation in the relative risk of GDM [53]. In contrast, a cross-sectional analysis involving two cohorts—the South Asian Birth Cohort (n = 976) and the Family Atherosclerosis Monitoring in Early Life (n = 581)—found that moderate red meat consumption was not significantly associated with GDM when compared to both low and high red meat intake [54]. These inconsistencies may be due to different dietary cultures and genetic factors. Notably, participants in this cross-sectional study reported higher plant protein intake, which may have counteracted some of the risks associated with red and processed meats. Additionally, distinct genetic variations associated with insulin resistance and glucose metabolism were identified in the South Asian population, both of which are significant risk factors for GDM [55].

Based on current research findings, there is evidence suggesting a positive association between red meat consumption and the risk of GDM. However, this association may be influenced by confounding factors such as overall dietary composition and genetic predispositions, which could modify the observed association.

### 2.7. Fish

Fish is often categorized alongside white meats in dietary contexts. Research indicated that adhering to a white meat protein pattern was associated with a higher risk of GDM compared to following a diet predominantly based on plant-based proteins, dairy, and eggs [56]. However, it is noteworthy that consuming sources of white meat, such as fish, was associated with a lower GDM risk relative to diets high in red meat [57]. A cohort study highlighted that the consumption of small oily fish, known for its high docosahexaenoic acid content and low levels of mercury, was associated with a decreased risk of GDM [58]. Oily fish served as an important source for humans to obtain marine *n*-3 long-chain polyunsaturated fatty acids [59]. These fatty acids are beneficial for placental growth and development and are associated with a lowering of the occurrence of adverse pregnancy outcomes in women with GDM [60].

Several RCTs have also elucidated the association between fish oil and GDM. For instance, a study that focused on obese and overweight pregnant women indicated that fish oil intake was not associated with GDM [61]. Interestingly, although the use of fish oil supplements did not influence fat mass or predict the incidence of GDM among overweight expectant mothers, it did affect the blood lipid profiles of non-GDM overweight or obese pregnant women [62,63]. However, another study found that obese or overweight individuals who consumed fish oil exhibited elevated *n*-3 long-chain polyunsaturated fatty acid levels within their serum. This condition was associated with a higher risk of GDM [64].

Based on existing evidence from cohort studies and RCTs, fish consumption is associated with a decreased risk of GDM. However, the effects of fish oil may vary among individuals, potentially posing an increased risk of GDM in expectant mothers with excess weight.

### 2.8. Poultry

There is a notable scarcity of studies specifically examining the relationship between poultry consumption and GDM. Most existing research integrates poultry within broader dietary pattern analyses, which limits the ability to draw specific conclusions about poultry consumption alone. For example, a retrospective study evaluated the dietary patterns of pregnant women approximately six months prior to GDM screening and found that the dietary patterns of women diagnosed with GDM exhibited more frequent poultry consumption [65]. Additionally, a cohort study focused on expectant mothers with twin gestations showed that a diet pattern predominantly consisting of poultry and fruits was not associated with an increased risk of GDM [66].

Currently, the available evidence does not sufficiently establish a clear association between poultry consumption and GDM. Therefore, more research is essential to explore the potential relationships between various types of poultry (such as chicken, turkey, and duck) and GDM.

### 2.9. Eggs

Eggs are recognized as a nutrient-dense food, providing essential components such as protein, cholesterol, and minerals [67]. While limited evidence suggests that egg intake throughout pregnancy is associated with a decreased risk of GDM [68], it is noteworthy that eggs are a primary source of dietary cholesterol [69]. For instance, a cohort study indicated an association between elevated cholesterol intake from eggs during pregnancy and an increased GDM risk [70]. Additionally, another cohort study emphasized that egg consumption was a significant driving factor associated with maternal dietary cholesterol intake and GDM [71]. In contrast, a case-control study found that egg consumption, as a source of protein, was not significantly associated with GDM [72].

The current evidence suggests a complex association between egg intake and GDM. However, inconsistencies across studies warrant cautious interpretations of this association.

### 2.10. Legumes

Several cohort studies have reported the association between legume consumption and GDM. For example, research focusing on women of reproductive age has shown that a higher intake of legumes was associated with a lower risk of GDM [32]. In addition, a study conducted in Japan found that the consumption of miso soup and natto was associated with a lower incidence of GDM both pre-conception and during early pregnancy [73]. Also, another study in China indicated that pregnant women who consumed higher amounts of soy products were associated with a lower incidence of GDM compared to those who did not consume soy products [74].

Furthermore, in a cohort study, participants were divided into two groups according to their daily soybean intake: a deficient group consuming less than 40 g per day and a control group consuming 40 g or more. The results indicated that the daily consumption of less than 40 g of soybeans was associated with a 2116-times increase in the risk of GDM [75]. Moreover, the consumption of soy protein by women with GDM has been shown to effectively lower the occurrence of neonatal hyperbilirubinemia and reduce hospitalization rates [76].

While much of the existing research has focused specifically on soy products, other related areas have also been investigated. Notably, the literature on other legumes, such as lentils, remains relatively sparse, and current studies have not identified significant associations between lentils and GDM [77].

In summary, the existing research indicates that the consumption of soy products is associated with a reduced risk of GDM and may also decrease the need for hospitalization in the offspring of women with GDM. Meanwhile, the association between lentils with GDM remains inconclusive. To date, no research has investigated the association between other types of legumes and GDM.

### 2.11. Nuts

Few studies have explored the association between nuts and GDM. Despite the high-fat content of nuts, research has shown an association between increased nut consumption and a reduced risk of GDM [78]. Moreover, a RCT study involving Chinese women aged 23–39 and 24–30 weeks into pregnancy demonstrated that pistachios served as an effective alternative to low-fat, high-carbohydrate foods, which are commonly recommended for managing GDM, and exhibited the potential for improving glycemic management in pregnant women affected by GDM [79]. Furthermore, peanuts are of particular interest because they contain resveratrol, a natural polyphenolic compound that was shown to be effective in promoting glucose metabolism and improving insulin sensitivity in certain animal models of GDM [80]. Additionally, a cohort study indicated that a moderate consumption of nuts was associated with better kidney health in women with GDM [81].

Although the number of studies is limited, evidence from cohort studies and RCTs suggests that nut intake is inversely associated with the risk of GDM. Additionally, consuming pistachios is associated with improved blood glucose management and kidney health, while resveratrol in peanuts may support glucose metabolism and insulin sensitivity, highlighting their potential association with GDM.

### 2.12. Added Fats

In addition to the previously mentioned fish oil, numerous studies have explored the association between olive oil and GDM, while limited research has examined the association of other types of added fats with GDM. RCTs and cohort studies documented that a diet rich in extra virgin olive oil (EVOO) had the potential to regulate maternal insulin resistance and prevent GDM [79,82]. For instance, an RCT showed that participants who adhered to a Mediterranean diet supplemented with EVOO exhibited a lower incidence of GDM compared to those who followed a standard diet with restricted fat intake [83]. Additionally, another RCT indicated that pregnant women with GDM who incorporated a diet high in EVOO experienced reductions in maternal triglyceride levels and weight gain [84].

Overall, the existing research suggests that a diet rich in EVOO may offer protective effects against GDM and positively influence triglyceride levels and weight management in expectant mothers with GDM.

### 2.13. Added Sugars

A high intake of added sugars in the diet has been associated with an increased risk of GDM [85,86,87]. The metabolism and absorption of added sugars could vary depending on their food sources and physical forms (liquid vs. solid) [88], which may have different associations with GDM. Three cohort studies primarily focused on sugar-sweetened beverages (SSBs), a primary contributor of added sugars in the diets of pregnant women [89,90,91]. A cross-sectional study indicated that approximately one-fifth of pregnant women and one-quarter of non-pregnant women of reproductive age had indicated consuming SSBs daily or more frequently [92]. Currently, there is a lack of research examining the relationship between other sources of added sugars and GDM. Moreover, a cohort study investigated the association between cola consumption and GDM and found that pregnant women whose cola consumption exceeded the median level (33.3 mL/day) exhibited a significantly raised risk of GDM in contrast to individuals with lower consumption [93].

Additionally, several studies elucidated the association between added sugars and the health status of the offspring of mothers affected by GDM. A lower intake of SSBs (≤1 serving per day) was associated with a reduced risk of obesity in children of GDM-affected mothers who received exclusive breastfeeding [94]. A cohort study in Norway found that the intake of sugar-sweetened carbonated soft drinks among pregnant women with GDM was inversely associated with newborn birth weight [95].

Research examining the association between added sugars and GDM remains limited. Existing evidence indicates a positive association between SSBs, such as cola, and GDM. Additionally, the consumption of SSBs is associated with the weight of children born to GDM-affected mothers.

## 3. Discussion

Based on this mini-review, a higher consumption of whole grains, fish, full-fat dairy products, and nuts is associated with a reduced risk of GDM, whereas red meat and sugary beverages are associated with an increased risk. A moderate intake of fruits is inversely and suggestively associated with a reduced risk of GDM. In contrast, the evidence for the association of tubers or starchy vegetables, vegetables, eggs, and poultry with the risk of GDM remains inconsistent. Furthermore, due to the limited research available on this topic, it can only be concluded that soybeans and olive oil consumption may be associated with a lower risk of GDM for legumes and added fats (Table 1).

The EAT-Lancet reference diet is a sustainable and healthy diet for global reference. At present, it has been studied in relation to cancer, cardiovascular disease, diabetes, and other fields, with most studies targeting the general population [22,96,97]. However, women of reproductive age represent a particularly vulnerable group, especially in resource-scarce areas, where they are at a greater risk of malnutrition and experience higher incidences of diet-related diseases [98]. While the EAT-Lancet reference diet provides intake recommendations for each food group, the evaluation and scoring methodologies used in different studies are inconsistent [99]. This inconsistency raises questions about the applicability of these scoring systems to women of reproductive age, highlighting the need for a tailored scoring system that accurately evaluates both human and planetary health.

The evidence of this mini-review is limited, which complicates efforts to draw robust conclusions regarding the relationship between the EAT-Lancet reference diet components and GDM. While there are a few studies investigating these associations, most have focused on subsets within each food group, which fails to provide a comprehensive understanding. For instance, the literature often highlights chicken and fish as sources of white meat but overlooks alternatives like duck and seafood. Likewise, nut studies primarily examine pistachios and peanuts, leaving other nuts unexplored, and while the link between olive oil and GDM is recognized, the role of other added fats, such as soybean oil, remains unexamined. Given the interconnectedness of the diet’s components, which mutually influence one another, further research is warranted to assess the overall association between the EAT-Lancet reference diet and GDM.

The studies included in this review were mainly cohort studies and cross-sectional studies that relied on participants’ self-reported diet data. This reliance may introduce measurement errors into the research [100]. Furthermore, variations in national and regional dietary cultures mean that the primary foods consumed within each group, as well as methods of preparation, can differ significantly. These factors may introduce biases when examining the relationship between entire food groups and GDM, potentially explaining the disparate findings across studies.

In the future, we should seek to identify the most representative scoring method for tailoring the EAT-Lancet reference diet specifically for women of reproductive age in designated regions. This strategy will help develop a targeted dietary pattern reflective of local economic, cultural, and dietary habits. Additionally, we can investigate the differences between this tailored dietary pattern and existing frameworks, such as the Mediterranean Diet and the DASH Diet, in the prevention of GDM. To enhance the accuracy of food intake assessments, we can incorporate advanced technological tools, such as digital tracking apps and post-event data analysis methods, to minimize inaccuracies in dietary reporting [100]. Once a strong research foundation is established regarding the EAT-Lancet dietary patterns and GDM, we can expand our investigation to elucidate the underlying mechanisms of this dietary pattern in association with GDM through a multi-omics approach (including genomics and metabolomics).

## 4. Conclusions

In conclusion, this mini-review evaluated the association of the EAT-Lancet reference diet components with GDM, addressed the limitations of the current research, and noted the need for a tailored scoring system for women of reproductive age. By summarizing these findings, the review provides researchers with areas that require further investigation and highlights future research directions.

## Figures and Tables

**Table 1:** Summary of relevant evidence retrieved from this review.

The EAT-Lancet Reference Diet Components	Conclusions from Available Evidence
Whole grains	Cohort studies indicate that whole grain consumption is inversely associated with the risk of GDM, with different processing methods potentially affecting this association.
Tubers or starchy vegetables	Inconsistencies among cohort studies hinder establishing a definitive association between tuber or starchy vegetable intake and GDM.
Fruits	Both RCTs and cohort studies indicate that moderate fruit intake can reduce the incidence of GDM. Furthermore, cohort studies suggest that fruit consumption is associated with improved outcomes for pregnant women with GDM.
vegetables	Cohort studies suggest that combined fruit and vegetable intake may reduce GDM risk, but few have investigated vegetable intake alone, leading to inconsistent evidence regarding its association with GDM.
Dairy foods	Cohort studies suggest that while full-fat dairy products may reduce GDM risk and cheese and yogurt are associated with a lower risk, the relationship between fat-free dairy products and GDM remains uncertain. RCT studies prove that vitamin D3-enriched yogurt is beneficial for insulin sensitivity in pregnant women with GDM.
Red meat	Cohort studies indicate a positive association between red meat intake and an increased risk of GDM, although this association may be masked by confounding factors.
Fish	Cohort studies and RCT evidence suggest that fish consumption is associated with a reduced risk of GDM, but the effects of fish oil may vary, potentially increasing GDM risk in overweight expectant mothers.
Poultry	Current evidence does not establish a clear association between poultry consumption and GDM.
Eggs	Cohort and case-control studies suggest that excessive egg consumption should be avoided. As a protein source, eggs show no association with GDM, but as a source of cholesterol, higher egg intake is positively associated with an increased risk of GDM.
Legumes	Cohort study results indicate that consuming at least 40 g of soybeans daily is associated with a reduced risk of GDM, while the relationship between other legumes, such as lentils, remains unclear.
Nuts	Cohort studies and RCTs suggest that a higher nut intake is associated with a lower risk of GDM, while consuming pistachios may benefit blood glucose management and improve kidney health in pregnant women with GDM.
Added fats	Cohort studies and RCTs suggest that a diet rich in extra virgin olive oil may protect against GDM and benefit the health of pregnant women with GDM.
Added sugars	Cohort studies indicate that SSBs are linked to an increased risk of GDM, with pregnant women consuming more than the median cola level (33.3 mL/day) showing a significantly higher risk. Additionally, SSB intake is associated with the weight of offspring from mothers with GDM.

Abbreviations: GDM: gestational diabetes mellitus; RCT: randomized controlled trial; SSB: sugar-sweetened beverage.
